# Integrated Immune and Molecular Profiling Identifies Prognostic Subgroups and Therapeutic Targets in Chondrosarcoma

**DOI:** 10.3390/ijms27042018

**Published:** 2026-02-20

**Authors:** Agnieszka E. Zając, Piotr Rutkowski, Anna Szumera-Ciećkiewicz, Jakub Piątkowski, Paweł Teterycz, Emanuela Palmerini, Aurélie Dutour, Justyna Tuziak-Klym, Michał Wągrodzki, Andrzej Pieńkowski, Andrzej Tysarowski, Marco Gambarotti, Giorgio Frega, Michela Pierini, Alberto Righi, Giovanna Magagnoli, Myriam Jean-Denis, Toni Ibrahim, Jean-Yves Blay, Paweł Golik, Anna M. Czarnecka

**Affiliations:** 1 Department of Soft Tissue/Bone Sarcoma and Melanoma, Maria Sklodowska-Curie National Research Institute of Oncology, Roentgena 5, 02-781 Warsaw, Poland; piotr.rutkowski@nio.gov.pl (P.R.); pawel.teterycz@nio.gov.pl (P.T.); andrzej.pienkowski@nio.gov.pl (A.P.); 2 Department of Pathology, Maria Sklodowska-Curie National Research Institute of Oncology, Roentgena 5, 02-781 Warsaw, Poland; anna.szumera-cieckiewicz@nio.gov.pl (A.S.-C.); justyna.tuziak-klym@nio.gov.pl (J.T.-K.); michal.wagrodzki@nio.gov.pl (M.W.); 3 Biobank, Maria Sklodowska-Curie National Research Institute of Oncology, Roentgena 5, 02-781 Warsaw, Poland; 4 Institute of Genetics and Biotechnology, Faculty of Biology, University of Warsaw, A. Pawińskiego 5A, 02-106 Warsaw, Poland; j.piatkowski@uw.edu.pl (J.P.); pgolik@igib.uw.edu.pl (P.G.); 5 Regional Center for Digital Medicine, Maria Sklodowska-Curie National Research Institute of Oncology, Roentgena 5, 02-781 Warsaw, Poland; 6 Osteoncology, Bone and Soft Tissue Sarcomas and Innovative Therapies Unit, IRCCS Istituto Ortopedico Rizzoli, Via Pupilli, 1, 40136 Bologna, Italy; emanuela.palmerini3@unibo.it (E.P.); giorgio.frega@ior.it (G.F.); michela.pierini@ior.it (M.P.); toni.ibrahim@ior.it (T.I.); 7 Sylvester Comprehensive Cancer Center, Miller School of Medicine, University of Miami, 1475 NW 12th Ave, Miami, FL 33136, USA; 8 Cell Death and Childhood Cancers Team, Centre de Recherche en Cancérologie de Lyon (CRCL)–UMR INSERM 1052–CNRS 5286 Centre Léon Bérard, 28 Rue Laennec, 69373 Lyon, France; aurelie.dutour@lyon.unicancer.fr; 9 Cancer Molecular and Genetic Diagnostics Laboratory, Maria Sklodowska-Curie National Research Institute of Oncology, Roentgena 5, 02-781 Warsaw, Poland; andrzej.tysarowski@nio.gov.pl; 10 Department of Pathology, IRCCS Istituto Ortopedico Rizzoli, Via di Barbiano 1/10, 40136 Bologna, Italy; marco.gambarotti@ior.it (M.G.); alberto.righi@ior.it (A.R.); giovanna.magagnoli@ior.it (G.M.); 11 Department of Biopathology, Centre Léon Bérard, 28 Rue Laennec, 69373 Lyon, France; myriam.jean-denis@lyon.unicancer.fr; 12 Centre Léon Bérard, 28 Rue Laennec, 69373 Lyon, France; jean-yves.blay@lyon.unicancer.fr; 13 Institute of Biochemistry and Biophysics, Polish Academy of Science, A. Pawińskiego 5A, 02-106 Warsaw, Poland

**Keywords:** chondrosarcoma, molecular profiling, immunological profiling, tumor microenvironment, immunotherapy

## Abstract

Chondrosarcoma (ChS) is a rare bone malignancy with heterogeneous behavior, the molecular and immunological background of which remains unknown. No effective systemic treatment for advanced ChS patients is available. The aim of this study was to develop an immune–mutational classification of ChS and to search for novel prognostic factors and molecular targets. We performed an immunological–molecular profiling of 99 patients diagnosed with primary ChS G1–G3 and dedifferentiated ChS. An expression of 20 immune response markers was assessed by IHC and targeted the next-generation sequencing of 409 genes was performed. Immunological and mutational profiles were correlated with overall survival using a multivariate LASSO-penalized Cox model. Three immunophenotypes were described—“cold” (IMP1), “hot” (IMP2), and “intermediate” (IMP3). IMP1 was the most prevalent in G1 cases, while IMP2 was the most prevalent in dedifferentiated cases. *IDH1*/*2* or *TP53* mutations were associated with high-grade ChS (FDR < 0.05). IMP2 was characterized by a higher number of immune infiltrates in the central region of the tumor (HR: 3.3; CI: 1.13–9.8; *p* < 0.05). *IDH1* mutations were present most often in IMP2 cases (HR: 3.8; CI: 1.75–8.1; *p* < 0.001). Tumor size, dedifferentiated subtype, *IDH1* mutation and the presence of IMP2 were identified as independent negative prognostic survival factors in ChS. An immune–mutational classification system for ChS patients was proposed, which may be used to identify those potentially suited for immunotherapy combined with IDH-mutant inhibitors in future research.

## 1. Introduction

Chondrosarcoma (ChS) is a rare primary malignant bone tumor, representing 20–30% of all bone sarcomas and ranking second in prevalence after osteosarcoma [1,2,3]. Despite representing only 1% of all malignancies, ChS exemplifies one of oncology’s most pressing therapeutic challenges. ChS encompasses a heterogeneous group of histological subtypes, each with distinct morphological features and prognostic outcomes [2]. The prognostic heterogeneity—from 99% 5-year survival in ChS grade 1 (G1) to less than 24% in dedifferentiated ChS (DDCS)—underscores the critical need for biomarker-driven patient stratification to guide therapeutic decisions and clinical trial enrollment [2,4].

The primary approach to managing ChS remains surgical excision with clear (R0) margins, as these tumors are highly resistant to standard chemotherapy and radiotherapy [5,6,7,8,9]. Currently, there is no specific treatment for locally advanced or unresectable ChS, since chemotherapy and targeted therapies typically have limited success [10,11,12]. Recent reports of responses to immune checkpoint inhibitors (ICIs) such as nivolumab and pembrolizumab in advanced ChS and DDCS—with objective response rates of 20–25% for DDCS—are promising but require validation in larger, more stratified cohorts [11,13,14,15].

Genomic profiling has revealed recurrent mutations in *IDH1*/*2*, enzymes central to the Krebs cycle, as well as alterations in PI3K-AKT, p53, and Hedgehog signaling pathways [2,16,17,18,19,20,21,22,23,24]. These insights offer potential targets for precision therapies. Despite low tumor mutation burden (TMB) and rare microsatellite instability (MSI), which typically predict poor immunotherapy response, the integration of multi-omic data may help identify actionable vulnerabilities. Personalized combination strategies targeting multiple oncogenic pathways are emerging as a viable approach [25].

The convergence of recent immunotherapy responses in selected ChS patients and advances in tumor immune profiling presents an opportunity to develop precision medicine approaches for this rare malignancy. However, the lack of validated biomarkers for patient selection remains a critical barrier to therapeutic progress. To overcome these challenges, this multicentric study aims to develop a classification system for ChS patients based on immunological and molecular profiles, enabling the identification of individuals who may benefit from immunotherapy, and discover new potential prognostic factors and molecular targets for future therapeutic strategies.

## 2. Results

### 2.1. Hierarchical Clustering of Immune Markers Identifies Three Prognostic Immunophenotypes in Chondrosarcoma

**Figure 1:**
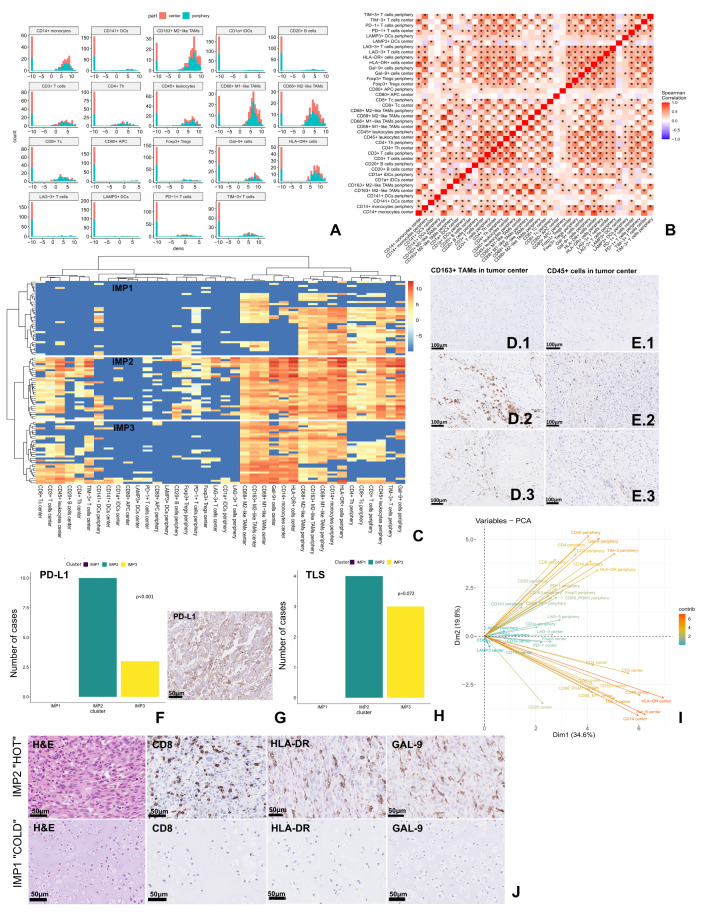
Immunological profiling of chondrosarcoma with immunophenotypes identification. (**A**) Distribution of individual immune markers in the central and peripheral regions of the tumor. (**B**) Correlation matrix between individual immune markers from different tumor regions. Asterisks indicate statistically significant results: * FDR < 0.05. (**C**) Heatmap showing three identified immunophenotypes, IMP1—”cold”, IMP2—”hot”, IMP3—”intermediate”, based on number and distribution of different immune cells. The density of immune cells was shown on a logarithmic scale from the lowest (dark blue) to the highest (red). (**D.1**–**E.3**) Immunohistochemical staining of CD163+ and CD45+ cells intratumorally in IMP1 (**D.1**,**E.1**), IMP2 (**D.2**,**E.2**) and IMP3 (**D.3**,**E.3**) groups. (**F**) Distribution of PD-L1 expression across immune clusters. Due to the fact that high expression of PD-L1 was observed only in DD, the PD-L1 expression level was not differentiated for comparative analyses between particular subgroups. (**G**) Example of immunohistochemical staining of PD-L1 in patient with IMP2. (**H**) Presence of TLS across immune clusters. (**I**) PCA of immunological markers’ importance in classifying across immune clusters. (**J**) Examples of immunohistochemical staining of selected markers of patients with IMP1 and IMP2. Abbreviations: APC—antigen-presenting cells, DC—dendritic cell, iDC—immature dendritic cell, Foxp3—Forkhead box P3, Gal-9—galectin 9, IMP—immunophenotype, LAG-3—lymphocyte activation gene-3, LAMP3—lysosome-associated membrane glycoprotein, PCA—principal component analysis, PD-1—programmed death receptor 1, PD-L1—programmed death ligand 1, TAM—tumor-associated macrophage, Tc—cytotoxic T cells, Th—helper T cells, TIL—tumor infiltrating lymphocyte, TIM-3—T cell immunoglobulin and mucin domain-3, TLS—tertiary lymphoid structures, Tregs—regulatory T cells.

A high density of HLA-DR+ antigen-presenting cells (APCs), CD14+ monocytes, CD68+ M1-like tumor-associated macrophages (TAMs), and CD163+ M2-like TAMs, as well as galectin 9 (Gal-9)+ cells was observed, with no differences between peripheral and central parts. CD68+ M2-like TAMs and TIM-3+ T cells were the second most frequently observed immune infiltrates, followed by helper and cytotoxic T cells (presented in Figure 1A, technical details of selected antibodies are presented in Table S1). The density of CD45+ leukocytes was significantly higher in the peripheral region of the tumor compared to the central region, which was also observed for programmed death receptor 1 (PD-1)+, CD3+, and CD4+ T cells, as well as CD141+ dendritic cells (DCs). The analysis of comparisons of densities of individual markers in the central and peripheral regions of the tumor is presented in the Table S2.

The strongest positive and significant correlation (rho > 0.9) was identified between the presence of M1 and M2-like TAMs (CD68+ and CD163+ cells) in the central part of the tumor. A strong positive correlation (rho > 0.7) was also observed for the expression of HLA-DR and CD45, CD14, Gal-9, TIM-3, or CD163 markers in the central and peripheral regions (Figure 1B). Furthermore, in the peripheral region of the tumor, both HLA-DR+ and TIM-3+ cells were correlated with the presence of PD-1+ cells. On the other hand, the presence of the CD14 marker strongly correlated with the infiltration of CD163+ M2-like TAMs and “exhausted” T cells (Gal-9+ or TIM-3+) in the central and peripheral regions of the tumor (Figure 1B).

Based on the clustering of immune data from both tumor regions, we identified three different immunophenotypes (IMPs): IMP1—a “cold” phenotype with the lowest density of total immune cells and most of the cells located in the peripheral tumor region (Figure 1C—IMP1,D.1,E.1,J-IMP1); IMP2—a “hot” phenotype with the highest number of immune cells in both the central and peripheral regions of the tumor, and the highest expression of HLA-DR+, CD163+, Gal-9+ and PD-1+, and CD8+ cells in the intratumoral region (Figure 1C—IMP2,D.2,E.2,J-IMP2); and IMP3—the “intermediate” phenotype with a lower number of immune cells compared to IMP2, but with the presence of various immune cells also intratumorally (Figure 1C—IMP3,D.3,E.3). IMP2 was also characterized by the highest expression of programmed death ligand 1 (PD-L1) (Figure 1F,G) and a more frequent presence of tertiary lymphoid structures (TLS) (Figure 1H), compared to IMP3. In the IMP1 cluster, neither PD-L1 expression nor TLS presence was observed. In addition, high expression of PD-L1 was detected mainly in the IMP2 group (5 of 6 cases). Of the greatest importance in the occurrence of individual IMPs was the presence of cells with HLA-DR, Gal-9, CD14, and CD45 expression in the central region of the tumor and Gal-9, CD45, and TIM-3 expression in the peripheral region (PCA in Figure 1I).

### 2.2. Grade-Dependent Immune Distribution Pattern

**Figure 2:**
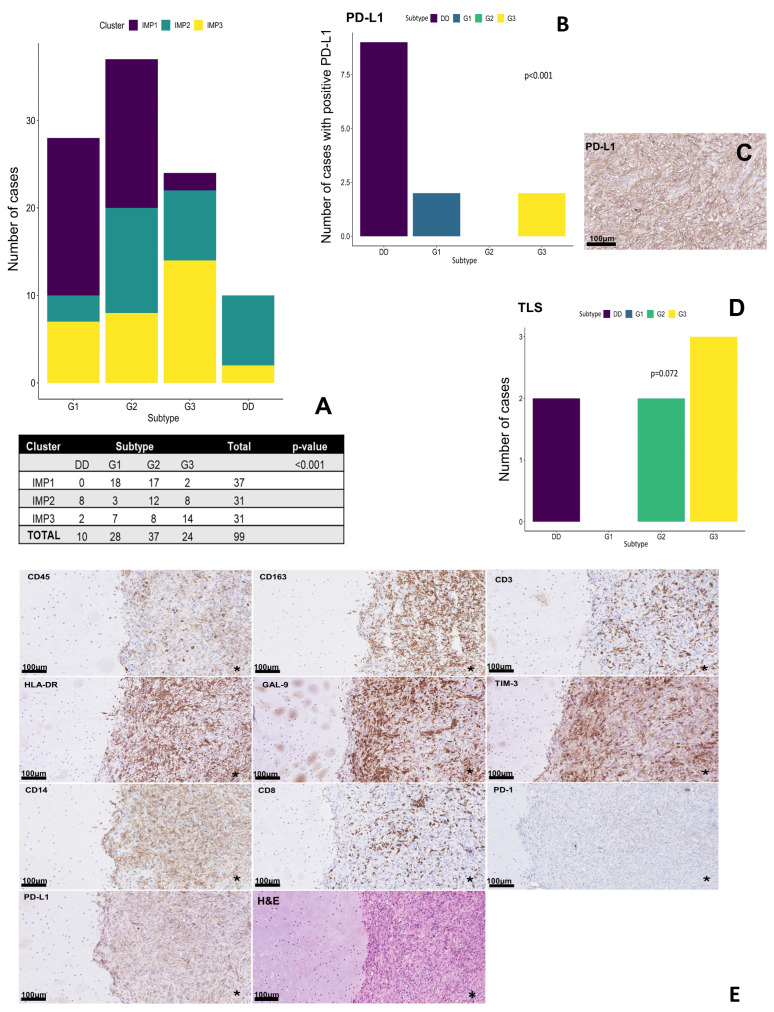
Grade-dependent immune distribution pattern. (**A**) Distribution of immune clusters across histological subtypes of chondrosarcoma with Fisher test comparison. (**B**) Distribution of PD-L1 expression across histological subtypes of chondrosarcoma. Due to the fact that high expression of PD-L1 was observed only in DD, the PD-L1 expression level was not differentiated for comparative analyses between particular subgroups. (**C**) Staining of PD-L1 in patient with dedifferentiated chondrosarcoma. (**D**) Presence of TLS across histological subtypes of chondrosarcoma. (**E**) Immunohistochemical staining of selected markers in dedifferentiated chondrosarcoma. Transition between low-grade and high-grade components was highlighted with asterisk (*). Abbreviations: DD—dedifferentiated chondrosarcoma, Gal-9—galectin 9, IMP—immunophenotype, PD-1—programmed death receptor 1, PD-L1—programmed death ligand 1, TIM-3—T cell immunoglobulin and mucin domain-3, TLS—tertiary lymphoid structures.

The presence of individual IMPs was different within the histological subtypes of ChS (Figure 2A). G1 ChS were characterized by a significant predominance of “cold” IMP1, while a similar proportion of the presence of IMP1 and “hot” IMP2 was observed in G2 ChS cases. In G3 cases, the proportion of IMP1 was lower compared to G1 and G2. DDCS was characterized by a higher number of immune cell infiltrates overall, especially in the differentiated component (Figure 2E), without the presence of IMP1. In addition, high expression of PD-L1 was observed only in DDCS, and its expression was significantly higher compared to other subtypes of ChS (Figure 2B,C). Interestingly, no PD-L1 expression was detected in G2 ChS (Figure 2B). On the other hand, TLS were observed only in high-grade ChS (G2/G3 and DDCS) (Figure 2D).

### 2.3. Molecular Profiling of Chondrosarcoma Revealed Significant Genetic Alteration Correlated with Aggressive Behavior

**Figure 3:**
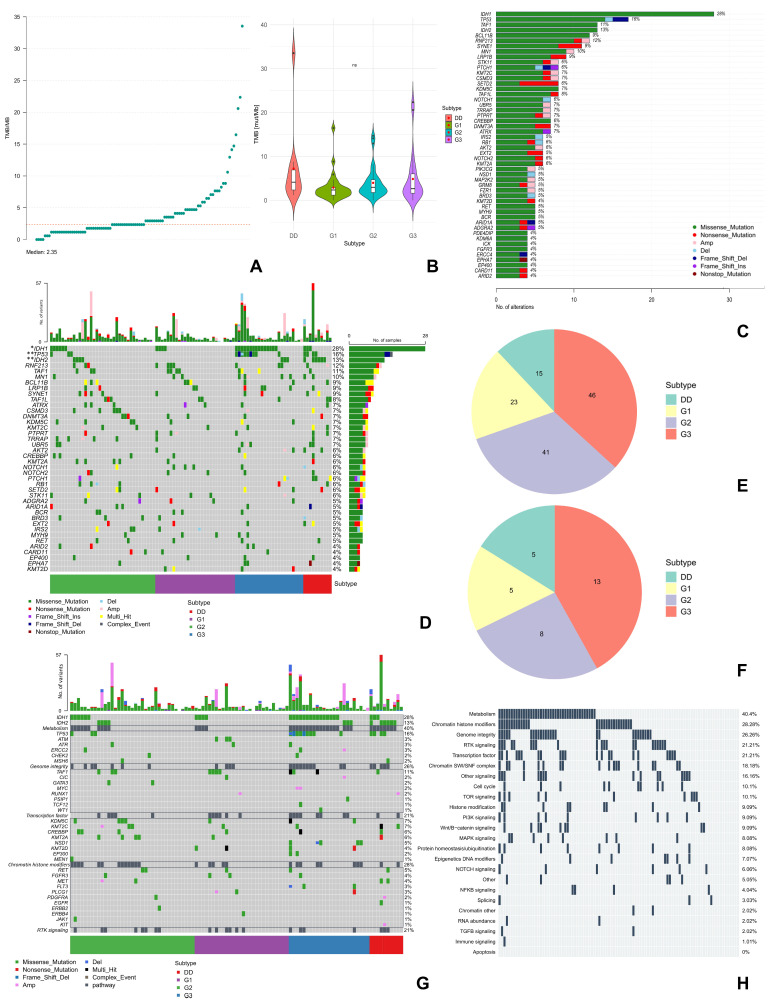
Molecular profiling of chondrosarcoma with grade-dependent molecular distribution pattern. (**A**) TMB values for individual patients with chondrosarcoma along with the median of the study population, marked with orange dotted line. (**B**) Box plots for TMB values according to the histologic subtype of chondrosarcoma. Red dots indicate their respective means. (**C**) Characterization of the 50 most common gene mutations across samples. Type of genetic variant is color-coded, values next to the bars identify the frequencies of each gene (%), the vertical axis presents the mutated genes, horizontal axis presents the number of variants per gene across all samples. (**D**) Summary of the 40 most common gene mutations across histological subtypes of chondrosarcoma with their frequency in the study population. The rows indicate mutated genes, the columns correspond to individual patients. The color legend below the chart shows the type of genetic variant. Gene mutations significantly different between histological subtypes are marked with asterisks: * FDR < 0.05; ** FDR < 0.01. (**E**) Distribution of amplifications across chondrosarcoma subtypes. (**F**) Distribution of large-scale deletions across chondrosarcoma subtypes. (**G**) Characterization of the top 5 deregulated pathways in the study population with their frequency among patients and including histological subtypes. (**H**) Characterization of all deregulated pathways and their frequency across the study population based on Bailey et al. [26]. Abbreviations: Amp—amplifications of chromosomal region, Complex event—identifies both single-nucleotide variants and genetic aberration, Del—large-scale deletion, DD—dedifferentiated chondrosarcoma, MAPK—mitogen-activated protein kinase, mTOR—mammalian target of rapamycin, Multi-hit—genes with more than one variant type, mut—mutated, NF-κB—nuclear factor kappa-light-chain-enhancer of activated B cells, NOTCH—neurogenic locus notch homolog protein, ns—statistically not significant, PI3K—phosphoinositide 3-kinases, SWI/SNF—SWItch/Sucrose Non-Fermentable, RTK—receptor tyrosine kinases, TCR—T Cell Receptor, TGFβ—transforming growth factor beta, TMB—tumor mutation burden.

All samples were microsatellite stable. The mean TMB in the study group was 4.21 mut/Mb (0 to 33.5 mut/Mb) with a median of 2.35 mut/Mb (Figure 3A). TMB was higher in high-grade ChS, although the differences were not statistically significant (Figure 3B). The median TMB for each subtype was 2.35; 2.95; 2.65, and 4.12 mut/Mb for ChS G1, G2, G3, and DDCS, respectively (Figure 3B).

We identified 709 potentially pathogenic and somatic single-nucleotide variants (SNVs) of genes in NGS. The summary of variants is presented in Figure S1A. In addition, we detected a total of 156 copy number variants (CNVs), which were detected in 26.3% of patients. Both amplifications and large-scale deletions were most frequently observed in the G3 ChS cases (Figure 3E,F).

In the study population, the most frequently mutated genes (present in at least 10% of the samples) were *IDH1*/*2* (41% of cases), *TP53* (16%), *RNF213* (12%), *TAF1* (11%), and *MN1* (10%) (Figure 3C and Figure S1A). However, only *IDH1* (FDR < 0.05), *IDH2* (FDR < 0.01), and *TP53* gene mutations (FDR < 0.01) were significantly different between histological subtypes of ChS (Figure 3D, Table S3—testing the differences in gene mutations across tumor subtypes). *IDH1* mutations were observed more frequently in ChS G2 and G3, whereas *TP53* mutations (including CNV deletions) were prevalent in G3 ChS. On the other hand, *IDH2* mutations were the most frequent in DDCS. None of the *IDH2* or *TP53* mutations was detected in G1 ChS (Figure 3D). Among the *IDH1* mutations we detected common for ChS variants [22,27,28]—R132/L/C/S/F/G/Q, but also atypical V71A variant (Figure S1C); while the *IDH2* mutations included R172/G/S/M variants, which has also been often reported [22,28,29] (Figure S1D). The *IDH1* mutations significantly co-occurred with *TP53* (*p* < 0.01) and *UBR5* (*p* < 0.01) gene mutations, whereas *IDH2* co-occurred with *PTCH1* mutations (*p* < 0.01) (Figure S1B).

Receptor tyrosine kinases (RTKs)-signaling and p53-related pathways, as well as metabolism and chromatin remodeling processes, were the most frequently (>20% of cases) deregulated processes in the study group (Figure 3G,H and Figure S1E,F). However, high-grade ChSs were more often associated with large-scale genetic changes (CNVs) in genes related to RTKs. For example, *FLT3* deletions and amplifications of *BRAF*, *MET*, *MAP2K2*, or *FGFR1* were not observed in G1 ChS, unlike in other subtypes (Figure 3G and Figure S1E), whereas *MYC* or *KIT* amplifications were observed only in G3 ChS or DDCS, respectively (Figure 3G). In addition, deregulations in pathways involved in cellular metabolism and genome integrity were predominant in high-grade ChS (especially G3 and DDCS) (Figure 3G).

### 2.4. Molecular Correlates Supporting the IMP Pattern

**Figure 4:**
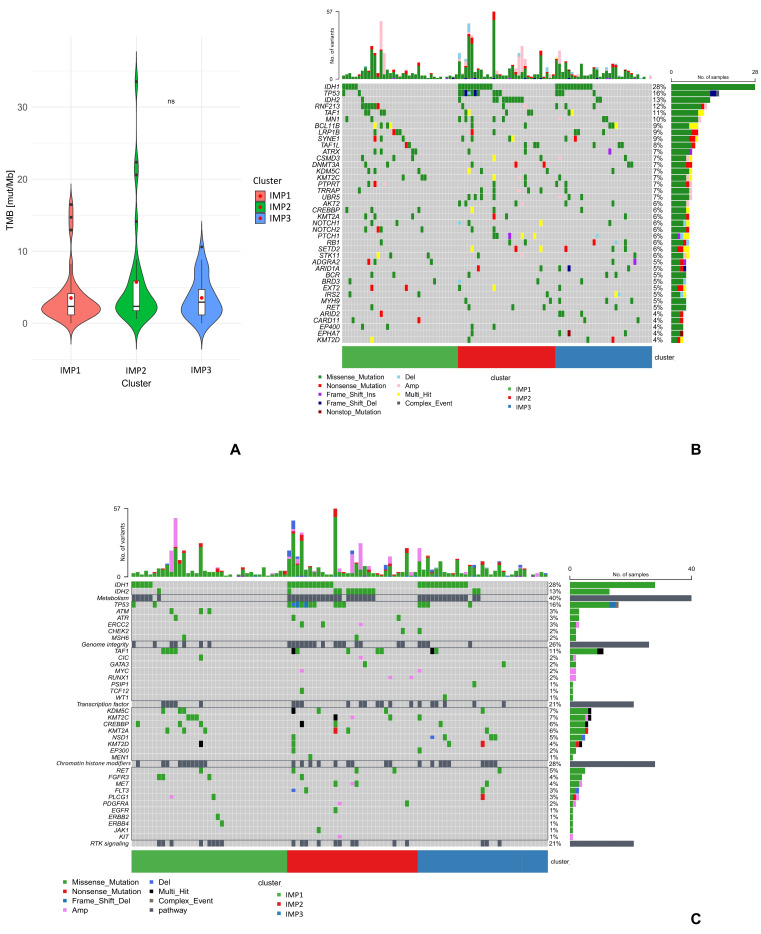
Molecular correlates supporting the IMP pattern. (**A**) Box plots for TMB values depending on the immunophenotype. The red dot indicates the mean. (**B**) Summary of the 40 most common gene mutations with their frequency in the study group, identified in each immunophenotype of chondrosarcoma. Mutated genes are in rows, individual patients are in columns. The color legend below the chart shows the type of genetic variant. (**C**) Characterization of the top 5 deregulated pathways in the study population with their frequency among patients and including immunological clusters. The classification was based on Bailey et al. [26] Abbreviations: Amp—amplifications of chromosomal region, Complex event—identifies both single-nucleotide variants and genetic aberration, Del—large-scale deletion, IMP—immunophenotype, Multi-hit—genes with more than one variant type, ns — statistically not significant, TMB—tumor mutation burden.

The integration of molecular and immunological data revealed that *IDH* mutations are preferentially associated with the immunosuppressive “hot” IMP2, providing a potential mechanistic link between metabolic reprogramming and immune dysfunction (Figure 4B). There were no significant differences in any of the mutated genes, nor TMB values, between the different IMPs (Table S3—testing the differences in gene mutations across immune clusters). However, the highest TMB values were observed in the IMP2 group with an average TMB of 5.73 mut/Mb, while for the other IMPs, the average TMB was around 3.5 mut/Mb (Figure 4A).

We noticed that the genetic profile varied among the different IMPs (Figure 4B). IMP2 showed the highest number of CNVs (e.g., *AKT2*, *STK11* amplifications, and *NOTCH1* deletions), as well as *IDH1*/*2* and *TP53* mutations. Thus, the IMP2 was associated with deregulation in both metabolic and p53-related pathways, as well as with the RTK-signaling pathway (e.g., amplifications in *MET*, *PDGFRA,* and *KIT* genes) (Figure 4C). IMP3 was characterized by *MN1* and *UBR5* amplifications, *PTCH1* and *RB1* CNV deletions, but also frequent *IDH1* mutations and a deregulated metabolic pathway. Mutations in the *PTCH1*, *RB1*, *AKT2,* or *UBR5* genes were not observed in the IMP1. In addition, *IDH* mutations were less frequent in this group (Figure 4B).

### 2.5. Clinical Implications and Immunological–Molecular System of Chondrosarcoma Profiling

**Figure 5:**
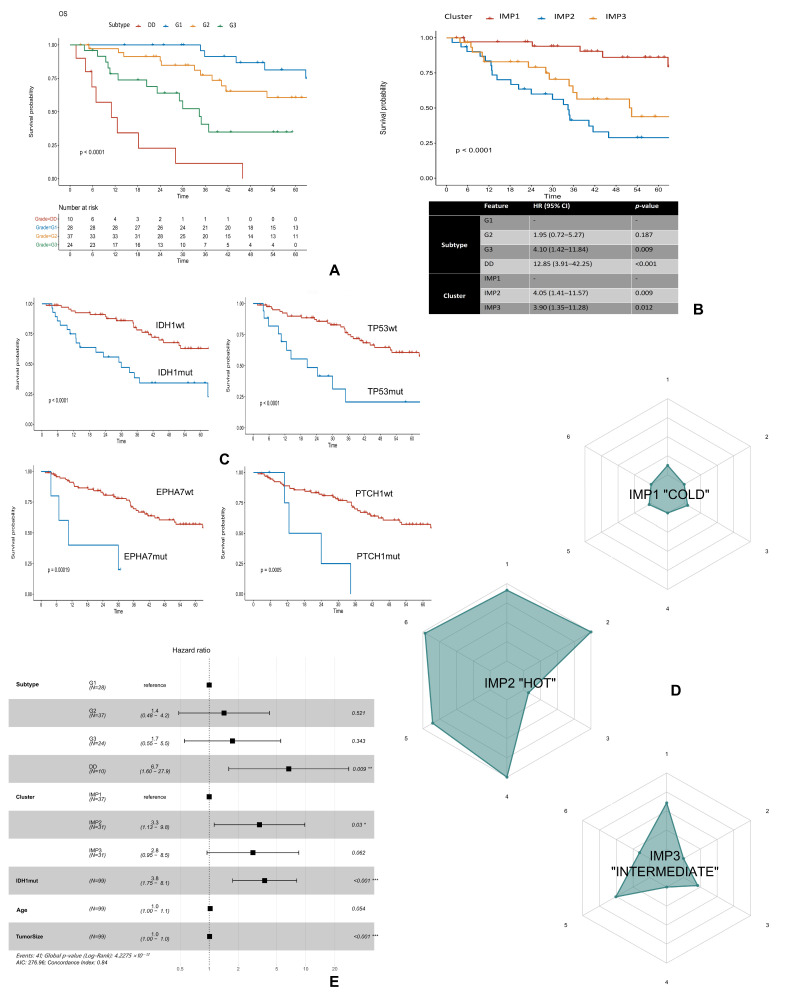
Clinical implications and immunological–molecular system of chondrosarcoma profiling. (**A**) Kaplan–Meier curves of overall survival depending on the histological subtype of chondrosarcoma. (**B**) Kaplan–Meier curves of overall survival in different immune clusters and table of multivariate Cox regression results. (**C**) Kaplan–Meier curves of overall survival in patients with and without mutations in the following genes: *IDH1*, *TP53*, *EPHA7* or *PTCH1*. (**D**) Radar diagrams representing the example of a patient with “cold”, “hot”, and “intermediate” IMPs. IMP2 represents patients who are candidates for immunotherapy and IDH1 inhibitor therapy with high levels of different immune cell infiltrates in general, as well as expression of PD-L1 and other ICPs. IMP1 represents the example of a patient being a poor candidate for immunotherapy with an absence of immune cells and ICPs. IMP3 represents a patient with an “intermediate” level of immune cell infiltrates and ICPs expression, but with frequent *IDH1* gene mutations instead. The axes represent: 1, the mean density of immune suppressive cells markers (Foxp3, CD163 and CD68 [KP1]); 2, the presence of IDH1 mutations; 3, the TMB status; 4, the presence of PD-L1 expression; 5, the mean density of the rest of immune markers from both the central and peripheral tumor region; and 6, the mean density of ICPs (TIM-3, LAG-3, Gal-9 and PD-1). (**E**) Forest plot presenting multivariate Cox regression results of the influence of clinical, molecular, and immunological factors on the overall survival of patients with chondrosarcoma. Asterisks indicate statistically significant results: * *p* < 0.05; ** *p* < 0.01; *** *p* < 0.001. Abbreviations: DD—dedifferentiated chondrosarcoma, ICPs—immune checkpoints, IDH1—isocitrate dehydrogenase, IMP—immunophenotype, wt—wild type, without mutation.

In the study population, the median overall survival (OS) was 64.6 (95% CI: 44.2—NR) months, while the median disease-free survival (DFS) was 32.1 (95% CI: 22–52.9) months. The 3-year OS rate was 91.3%, 77.3, 40.7%, and 11.4% for patients with diagnosed G1, G2, G3 ChS, and DDCS, respectively (Figure 5A).

In multivariate analysis, histological subtype and tumor size had a negative impact on patients’ OS and DFS. In addition, older age correlated with poor OS, while lower limb tumor location correlated with poor DFS (Table S4).

We identified three distinct IMPs with significant prognostic implications. Patients with the “hot” IMP2 were paradoxically associated with the shortest OS. The 5-year OS in this group of patients was only 29%, while for the IMP3 and IMP1 it was 44% and 86% (*p* < 0.05), respectively (Figure 5B).

Univariate Cox regression revealed four gene mutations that could negatively impact the OS of ChS patients, including *IDH1*, *TP53*, *EPHA7,* and *PTCH1* genes with FDR < 0.05 (Table S5). The 5-year OS of patients with *TP53* mutations was only 21%, while for patients without these mutations, the OS was 61%. For patients with and without *IDH1* mutations, the 5-year OS was 34% and 63%, respectively. Patients with *EPHA7* or *PTCH1* gene mutations did not achieve the 3-year OS, while the 5-year survival rates for patients without these alterations were 57% (Figure 5C).

Constructed immunograms revealed that patients with IMP2 and the poorest prognosis had more immune cells in general (Figure S2), including immune suppressive cells, e.g., CD163+ TAMs (Figure 5D-IMP2, axis 1). In addition, these patients strongly expressed different ICPs, e.g., PD-L1, Gal-9 (Figure 5D-IMP2, axis 6) and more frequently displayed *IDH1* mutations, unlike patients with IMP1 (Figure 5D-IMP1, axis 2). Patients with IMP3 as well as poor prognostics had some immune cell infiltrates but with frequent *IDH1* mutations (Figure 5D-IMP3).

Final multiparametric analysis showed that in addition to tumor size and histologic subtype of ChS, the presence of an IMP2 (HR: 3.3, CI: 1.13–9.8, *p* < 0.05) and *IDH1* mutations (HR: 3.8, CI: 1.75–8.1, *p* < 0.001) were independent negative prognostic factors (Figure 5E). Among all analyzed immune markers, only the occurrence of specific IMP were significant in the final analysis, opposite to individual markers.

## 3. Discussion

This study presents the first comprehensive immuno–molecular classification of chondrosarcoma and explores the relationships among immune microenvironmental profiles, molecular alterations, and clinical outcomes. We also indicate new directions for further ChS research.

We identified three different IMPs: IMP1 (“cold”), IMP2 (“hot”), and IMP3 (“intermediate”). Interestingly, IMP2 and *IDH1* mutations were independent poor prognostic factors in ChS, and we believe that patients displaying those features can potentially benefit from immunotherapy and targeted therapies. The poorer prognosis seen in IMP2 (“hot”) tumors may be linked to an immune-exhausted phenotype. Although IMP2 showed the highest density of immune infiltrates, these were mainly tumor-associated macrophages expressing CD14, HLA-DR, and CD163, as well as Gal-9-positive cells. While M2 polarization was not predominant, TAMs overall could contribute to tumor progression and immunosuppression [30]. Therefore, this IMP can be classified as an “immune-infiltrated but functionally suppressed” IMP, which may explain the poorest prognosis in this group of patients. The significant co-expression of TIM-3 and PD-1 at the tumor periphery suggests the presence of TIM-3+PD-1+ T cells, consistent with “exhausted” CD8+ T cells previously reported in ChS [31]. Additionally, PD-L1 expression was correlated with IMP2/IMP3 and poor survival, confirming earlier findings that identify PD-L1 as a negative prognostic factor in ChS [32,33]. However, since PD-L1 expression was mainly observed in DDCS and it was observed in a low number of patients in total, these results should be interpreted with caution and regarded as exploratory results rather than conclusive. Meanwhile, Galectin-9 expression, although not previously reported in ChS, is a known immunosuppressive mediator in osteosarcoma. TIM-3/Gal-9 interactions can suppress Th1 cell activity and promote regulatory T cell expansion [34]. Gal-9 may also induce apoptosis of CD8+ T cells or influence macrophage polarization toward M2-like phenotypes [35,36]. Our findings suggest that Gal-9 contributes to a dysfunctional immune environment in ChS, potentially explaining the discrepancy between HLA-DR expression and ineffective antigen presentation. The presence of antigen-presenting cells without a corresponding cytotoxic response indicates defective immune activation in ChS, resulting in poor survival. Therefore, Gal-9 could serve as a promising prognostic biomarker and therapeutic target in ChS.

The presence of an immunosuppressive environment in ChS has also been suggested in some other studies. In the analysis of 21 cases of conventional ChS (G1–G3), three immune clusters were described: “granulocytic-myeloid-derived suppressor cell (G-MDSC)-dominant”, “immune-exhausted”, and “immune-desert” phenotypes [31]. In addition, Richert et al. [37] observed the presence of several markers suggestive of an immunosuppressive TME, such as the expression of PD-L1 in DDCS, and the presence of TAMs (CD68+ or CD163+) and TILs in DDCS and conventional ChS [37]. Additionally, a study involving over 500 soft tissue sarcomas identified immune phenotypes (“hot,” “moderate,” “cold”) like our findings, with the immunosuppressive ‘hot’ subtype showing the shortest progression-free survival (PFS) [38].

The presented work in this study, that increased immune cell infiltration of DDCS compared to conventional ChS, has been observed in several other studies [37,39], as well as higher immunogenicity in high-grade ChS (G2/G3) compared to G1 ChS [31,40]. The increased immunogenicity in high-grade ChS may be related to the morphological changes, that is, a reduction in the cartilaginous matrix (in G2/G3 ChS) or the dedifferentiation of chondrocytes in DDCS [2,41,42], which may lead to facilitated infiltration of immune cells in these ChS subtypes.

In our study, the processes related to epigenetic processes, e.g., histone chromatin modifiers, were deregulated in approximately one-third of patients. Histone modifications, including methylation, can be associated with *IDH* mutations and overproduction of the oncometabolite 2-hydroxyglutarate (2-HG), which can further lead to the silencing of gene expression [43,44,45]. As previously reported, aberrant epigenetic regulation, mediated by IDH mutations, appears to be integral to ChS pathogenesis [46,47]. Molecular profiling revealed frequent *IDH1*/*2* mutations in high-grade tumors (G3 and DDCS), supporting their role as early driver events promoting progression, which can be responsible for the progression from low-grade to high-grade, particularly toward DDCS [29,48]. On the other hand, epigenetic studies showed that conventional ChS and DDCS have different methylation profiles, which can depend on the presence of *IDH1*/*2* mutations. This may cause the *de novo* development of a more malignant form of ChS [27,48,49].

In multivariate analysis, *IDH1* mutations were confirmed as having independent prognostic significance. Although previous studies yielded inconsistent survival data [28,29,44,50,51], our results and a recent analysis of 488 ChS patients (HR = 1.90; 95% CI: 1.06–3.42; *p* = 0.03) confirm the adverse prognostic role of *IDH1*/*2* mutations in ChS [52]. *IDH1*/*2* mutations and 2-HG overproduction are related to many cellular processes that could potentially affect the survival of ChS patients. Mechanistically, *IDH* mutations and 2-HG accumulation disrupt NADP^+^/NADPH balance, elevate reactive oxygen species (ROS), and enhance DNA damage. They may also activate mTOR signaling independently of PI3K/PTEN, or lead to global hypermethylation [27,44,45,53]. On the other hand, *IDH1* mutations could also influence the TME of ChS. Mutations in this gene predominantly affected patients with IMP2 and IMP3. The positive correlation between the *IDH1* mutation and infiltration of T cells, DCs, and G-MDSC has already been found in ChS patients [31]. The 2-HG-mediated alteration of the extracellular matrix (ECM) and immune cell phenotype may impair immune surveillance and facilitate tumor escape [44,54]. Analogous mechanisms in gliomas demonstrate that *IDH1* mutations can suppress NK and T cell function, promoting immunosuppression [55]. The 2-HG, particularly the D-enantiomer produced by mutated IDH1/2 enzymes, acts as an oncometabolite that drives tumor progression by reprogramming the TME, leading to T cell exhaustion and suppression of NK cells. Moreover, the hypermethylated state caused by 2-HG overproduction inhibits cellular differentiation, trapping tumor cells in an undifferentiated, stem-like state [56]. Therefore, *IDH* mutation can finally promote a pro-tumorigenic, immunosuppressive environment. These findings imply a dual oncogenic role for *IDH1* mutations and 2-HG-mediated metabolic and epigenetic pathways—driving tumor progression and modulating immune evasion in ChS.

Moreover, *TP53* and *RB1* mutations, found in high-grade tumors, especially in G3 ChS, confirm earlier reports designating p53 pathway alterations as late events in ChS progression [22,57,58,59]. In addition, the presence of large-scale mutations in genes encoding kinases and growth factor receptors in G2/G3 ChS and DDCS suggests an important role of RTK-signaling pathways, including RTK-Ras-extracellular signal-regulated kinase (ERK) and/or PI3K-Akt [60,61], in the progression of these tumors.

The evaluation of *EPHA7* and *PTCH1* mutations as prognostic factors in the OS of ChS seems to be unique for the present study. The *EPHA7*, a member of the ephrin RTK family, participates in PI3K–AKT-dependent tumorigenic pathways [62,63]; whereas *PTCH1* encodes a key component of the Hedgehog pathway [24]. Their association with poor prognosis, particularly in high-grade ChS, highlights their potential as novel molecular markers with potential clinical implications. These findings also suggest that Hedgehog signaling may influence both tumorigenesis and subsequent tumor progression [19].

Our multiparametric study integrated immunophenotypic and genomic data from nearly 100 ChS patients (G1–G3, DDCS) and demonstrated that ChS classification based on immune–molecular profiles is feasible and clinically relevant. Both the histological grade and molecular profile shape the ChS immunological landscape, with the “hot” IMP2 phenotype and *IDH1* mutations independently predicting poor survival. Our study confirms the potential for precision immuno-oncology approaches in ChS. Patients harboring both biomarkers may benefit from dual-targeted strategies combining immune checkpoint blockade (e.g., TIM-3/Gal-9 inhibition) with IDH-mutant-targeted therapy. Although early clinical trials of PD-1/PD-L1 inhibitors in ChS yielded limited benefit [10,11,12]; our results indicate that alternative checkpoints (Gal-9, TIM-3) warrant exploration as therapeutic targets.

In conclusion, our study provides a novel immune–molecular taxonomy of ChS, integrating immune phenotypes with mutational signatures. The identification of a poor-prognosis subgroup characterized by “hot” IMP2 and *IDH1* mutations may support the rationale for combined immunotherapy and metabolic-targeted interventions. It may also support the use of personalized therapeutic approaches in patients with ChS. The combination of ICP inhibitors and targeted therapies against TME modulating factors is currently being tested in numerous clinical trials and appears promising in the treatment of solid tumors [64]. The results presented in this paper suggest that Gal-9/TIM-3 and IDH1 inhibitors may be a promising direction for developing a future therapy for these tumors. However, further investigation into the molecular crosstalk between *IDH1* mutations, epigenetic dysregulation, and immune suppression will be essential for developing effective personalized treatment strategies in ChS. Importantly, the proposed immune classification is exploratory and requires validation in independent cohorts. Further mechanistic studies, including the development of appropriate in vitro models, will be necessary to assess the biological relevance of these associations and to evaluate potential therapeutic strategies.

## 4. Materials and Methods

### 4.1. Patient Recruitment and Study Design

We retrospectively enrolled 147 patients with histologically confirmed primary ChS, who were treated at three high-volume centers in Maria Sklodowska-Curie National Research Institute of Oncology (Warsaw, Poland), IRCCS Istituto Ortopedico Rizzoli (Bologna, Italy), and Centre Léon Bérard (Lyon, France). Histological diagnosis was performed by expert sarcoma pathologists. Patients included in the study were diagnosed between 2010 and 2022 and were radically treated with surgical resection. The patients were qualified based on the presence of complete clinical data with at least one year of follow-up and the presence of good-quality resection material. The exclusion criteria were previous neoadjuvant chemotherapy or radiotherapy, subtotal resection, or a lack of a sample from the primary tumor site.

A total of 181 patients were screened, and 99 patients diagnosed with different subtypes of ChS (28 G1, 37 G2, 24 G3 ChS, and 10 DDCS), with complete immunological and molecular data included in the final multiparametric analysis (Figure S3A,B). The characteristics of 99 patients are presented in the Table S6. Samples were collected from formalin-fixed paraffin-embedded (FFPE) tissue, which were then used for targeted next-generation sequencing (NGS) of 409 genes, immunohistochemistry (IHC) of 20 markers (from central and peripheral tumor region) for both the immune and tumor cells, and MSI analyses.

### 4.2. Clinical Data Evaluation

We analyzed gender, tumor size, age, pathological fracture, tumor site, surgical margin, and histological subtype using univariate and multivariate Cox regression to evaluate their impact on patient survival (OS, DFS). Kaplan–Meier curves were generated, and statistical differences between groups were assessed with the log-rank test. OS was defined from diagnosis to death or last follow-up; DFS spanned from radical resection to recurrence, metastasis, death, or follow-up.

### 4.3. Tissue Microarrays Preparation and Immunohistochemistry

The tissues were fixed in buffered 10% formalin (fixation time was no longer than 48 h). The tumor content in the tissue samples was at least 80%, as assessed by a pathologist. The material did not contain bone marrow components. Our selection criteria ensured minimal interference from non-tumor cells. The material was selected as much as possible without treatment with a decalcifying agent. TMAs were constructed from FFPE tissue blocks, including two representative 2.0 mm cores from the central and peripheral regions of the tumor. In each TMA block, the liver, tonsil, kidney, and testis were used as control tissues. In this way, 8 TMAs were prepared for further IHC staining.

IHC staining was performed on four-μm-thick tissue sections using an automated stainers Autostainer Link 48 automated device (Agilent Technologies, Inc., Santa Clara, CA, USA) or Ventana Benchmark Ultra (Ventana Medical System Inc., Oro Valley, AZ, USA). We selected 20 markers for IHC evaluation, including leukocytes (CD45), T cells (CD3, CD4, and CD8), Tregs (Foxp3), B cells (CD20), M1-like TAMs (CD68 clone [PG-M1]), M2-like TAMs (CD163 and CD68 clone [KP1]), monocytes (CD14), APCs (CD80 and HLA-DR), iDCs (CD1a), DCs (CD141 and LAMP3), and ICPs (PD-1, TIM-3, LAG-3, Gal-9, and PD-L1). Details and dilutions for each antibody used are presented in Table S1. The finished slides were scanned, and the image analysis was performed using QuPath software version 0.4.3 [65] and manually verified. PD-L1 expression was evaluated according to the literature knowledge as the percentage of positive cells on a scale: 0, no expression: <1% of positive cells; 1, low expression: 1–30% of positive cells; and 2, high expression: ≥30% of positive cells) [66]. For other markers, the number of positive cells per area (mm^2^) was assessed [67,68]. The presence of TLS was evaluated based on the positive expression of CD3 (T cells), CD20 (B-cells), and LAMP3 (DCs) [69] markers and confirmed by the presence of the organized aggregates of immune cells in H&E staining.

### 4.4. Determination of Immunophenotypes (IMPs)

Hierarchical clustering based on Euclidean distance and Ward’s D2 method was used to determine IMPs. The optimal number of clusters (three) was determined using between 30 indexes as implemented in NbClust R package [70]. To assess the internal validity and stability of the IMPs, bootstrap resampling of the immunohistochemical dataset (*n* = 99) was performed. In each of 1000 bootstrap iterations, cases were sampled with replacement. Cluster assignments from each bootstrap iteration were compared with the original clustering solution using the adjusted Rand index (ARI) (Table S7, Figure S4).

The contribution of individual immune markers across individual IMPs was assessed using principal components analysis (PCA).

### 4.5. DNA Isolation

DNA was extracted from FFPE tissue (10 µm thick tissue slides) using Biosystems™ MagMAX™ FFPE DNA/RNA Ultra kit (Thermo Fisher Scientific Inc., Waltham, MA, USA), following the manufacturer’s protocol (with the overnight incubation variant at 57 °C). Samples were stored at −20 °C. DNA concentrations were measured fluorescently with the Qubit dsDNA HS Assay Kit (Thermo Fisher Scientific Inc.) using a Qubit 2.0 fluorometer (Thermo Fisher Scientific Inc.) and following the manufacturer’s instructions. To assess the purity of the nucleic acids, we used a NanoDrop™ 2000 spectrophotometer (Thermo Fisher Scientific Inc.) and followed the manufacturer’s recommendations.

### 4.6. DNA Library Preparation and Sequencing with Targeted Gene Panel

DNA libraries were prepared using the Ion AmpliSeq™ Library Kit Plus (Thermo Fisher Scientific Inc.) and the Oncomine™ Tumor Mutation Load Assay genetic panel of 409 genes and 1.65 Mb of genomic space (Thermo Fisher Scientific Inc.). To avoid deamination related to FFPE tissue processing [71], we preventively used digestion with the non-heat-stable enzyme UDG (Thermo Fisher Scientific Inc.) for all samples as the first step in library preparation, according to the manufacturer’s recommendation, and followed the instructions in the protocol in further library preparation steps. We used the Ion P1 Adapter and Ion Xpress™ Barcode Adapters 33–48 Kit (Thermo Fisher Scientific Inc.) for the ligation step. The prepared libraries were purified using magnetic beads (Agencourt AMPure XP, Beckman Coulter Inc., Brea, CA, USA) following the manufacturer’s protocol, eluted with low TE buffer (Thermo Fisher Scientific Inc.), and stored at −20 °C. The concentrations of DNA libraries were measured using Qubit 2.0 and the molarity of each library was determined with the Agilent High Sensitivity DNA Kit (Agilent Technologies, Inc.) using the Bioanalyzer 2100 (Agilent Technologies, Inc.). The final libraries were pooled (in up to four per chip) in equimolar concentrations and used for automatic template preparation on the Ion Chef™ instrument (Thermo Fisher Scientific Inc.) using the Ion PI™ Hi-Q™ Chef Kit (Thermo Fisher Scientific Inc.) and the Ion 540™ Chips (Thermo Fisher Scientific Inc.). We used the Ion Proton™ sequencer (Thermo Fisher Scientific Inc.) for sequencing, and followed the manufacturer’s instructions to perform the sequencing reaction, using dedicated reagent kits and consumables: Ion PI™ Hi-Q™ Sequencing 200 Kit, Ion PI™, Ion PI™ Sequencing Nucleotides, and Ion Proton™ Sequencing Supplies (Thermo Fisher Scientific Inc.).

### 4.7. Bioinformatics Analysis of Data from Sequencing

Bioinformatics analysis of the sequencing data was performed using three different approaches:

1. Raw sequencing data were pre-processed on the sequencer using dedicated software—Torrent Suite^TM^ (TS) software version 5.14.0 (Thermo Fisher Scientific Inc.)—which included adapter sequences trimming, removing poor signal reads, and assigning the reads to a given sample based on the barcode. The processed reads were mapped to the reference genome hg19 (Homo sapiens) and adjusted to the specific amplicon target regions, based on the BED file provided by the manufacturer. Next, we performed coverage analysis (v5.12.0.0) and variant calling (v5.12.0.2) using plug-ins from TS and under the default low-stringency settings to call somatic variants (v5.12.0.2 (552)). The following criteria were used for qualitative evaluation: loading of the chip ≥ 80%, polyclonality ≤ 30%, uniformity ≥ 80%, at least 10 million mapped reads, and an average depth of coverage ≥ 1000. In some cases, with slightly fewer reads or coverage, reads from two independent sequencing runs for 1 sample were merged to get a higher number of mapped reads. Finally, we downloaded FASTQ, BAM, and VCF files from TS.
2. Next, the FASTQ files from the TS server were mapped to the hg19 reference genome using the Burrows–Wheeler Alignment (BWA-MEM) algorithm and analyzed with the Genome Analysis Toolkit (GATK, v4.2.2.0) [72] with the Base Quality Score Recalibration step. The mutect2 tool [73] in GATK was used to identify somatic variants. Population data from the Genome Aggregation Database (gnomAD) and the Panel of Normals (PoN) from GATK were used to filter out germline variants. The VCF files were then normalized, left-aligned and filtered based on the following parameters: for single-nucleotide polymorphism (SNP) or multiple nucleotide polymorphism (MNP): read depth (DP) ≥ 15, allele frequency (AF) ≥ 0.015, tumor log-odd score (TLOD) ≥ 14; for INDELs: DP ≥ 70, AF range: 0.35–0.55 [74,75].
3. Finally, we used BAM files from TS to perform variant calling and obtain VCF files using the commercial software Ion Reporter^TM^ (IR) (v5.20) with Oncomine Tumor Mutation Load—w3.4—DNA—single-sample plug-in. Samples with a deamination score < 10 were qualified for further analysis. For a deamination score in the range of 10–25 and/or TMB (calculated based on IR software v.5.20) in the range of 11–50 mut/Mb, we applied stricter conditions for filtering out variants for 6 samples by increasing the minimum AF from the default value of 0.05 to 0.1, according to the manufacturer’s recommendation [76]. For those samples, the AF value was also changed during analysis with GATK from 0.015 to 0.75 for SNP and MNP variants (to obtain the same ratio of the number of filtered variants before and after changing the AF parameter). We used “Oncomine Extended (5.14)” filtration for all samples, except the ones with a higher deamination score, for which we applied the default filtering “Oncomine Variants (5.10)”.

We then merged all three VCF files to obtain the most comprehensive identification of variants. For this purpose, we used the intersection function of bcftools [77] to merge VCF from GATK and TS, which allowed for an optimal ratio of SNP variants to INDELs, and then manually added variants from IR to the VCF file. The merged VCF files were annotated using the FUNCtional annOTATOR (Funcotator) from GATK version 4.2.2.0.

To validate the methodology and detect potential artifacts due to the lack of paired samples from the patients, a control group was established from a “healthy” population. For this purpose, nine available tissues (5 derived from FFPE tissues: skin, lymph nodes, or lungs, and 4 isolated from the blood of “healthy” individuals) were subjected to the same procedure of DNA library preparation, sequencing, and subsequent data analysis as described above. A VCF file containing a pool of variants from all “healthy” tissue samples was used to filter out the common variants from both files, which were potential germline variants, non-pathogenic variants, or artifacts. Finally, we performed post-variant filtration based on the following parameters: AF ≥ 0.05 and DP ≥ 50 [74,78,79]. Recurring putative artifact variants were additionally tested with Sanger sequencing.

Annotation of variants was also performed using the Ensembl Variant Effect Predictor (VEP) version 110.1 [80], while the pathogenicity of variants was predicted using Sorting Intolerant From Tolerant (SIFT) [81], Polymorphism Phenotyping v2 (PolyPhen-2) [82], and AlphaMissense [83] predictors. Variants identified by VEP as “low” IMPACT and “benign” according to the ClinVar database [84] were filtered out. In addition, variants were selected based on classification as “deleterious/pathogenic” by SIFT and Poly-Phen-2 or by AlphaMissense. Finally, variants that have not yet been described in the Single-Nucleotide Polymorphism Database population (dbSNP) [85] were manually reviewed for additional verification of the occurrence of potential artifacts (especially for INDELs) using Golden Helix GenomeBrowse^®^ version 3.0.0 software (reference sequence GRCH37 g1k, 1000 Genomes). Variants present in the “1000 Genomes” database [86] with AF > 0.05, with less than 5 reads in total and less than 2 reads in each strand, were excluded from the analysis [78,79].

### 4.8. Tumor Mutation Burden Analysis and Visualization of NGS Data

The TMB was calculated as the number of final variants (excluding CNVs) per size of the gene panel: 1.7 Mb. The Maftools R package (v2.18.0) [87] was used to visualize the data obtained from NGS. Functional analysis of deregulated genes was performed based on the data available from two different papers [26,88] using the Maftools package.

### 4.9. Copy Number Variants Detection

The PureCN R package version 2.0.2 [89] was used to identify copy number variants. Variant calling was performed based on BAMs obtained from GATK (v4.2.2.0) and using Mutect2 (v4.2.2.0). The previously described pool of nine “healthy” samples was used for the normalization.

### 4.10. Microsatellite Instability Evaluation

To assess MSI in the study population, we used the automated Idylla^TM^ MSI Test (Biocartis US, Inc., Raritan, NJ, USA), which is based on the qPCR of seven MSI gene loci, *ACVR2A*, *BTBD7*, *DIDO1*, *MRE11*, *RYR3*, *SEC31A,* and *SULF2*, following the manufacturer’s instructions. For this purpose, we used 1–2 FFPE tissue slides of 10 µm thickness. The results were analyzed using the Idylla^TM^ system (v26.0).

### 4.11. Evaluation of Immunological and Genetic Prognostic Factors and Multivariate Analysis

Univariate and multivariate analyses of the effect of genetic and immunologic factors on patients’ OS were performed using Cox regression. To select significant factors with an impact on patients’ OS we have incorporated all clinical, immunohistopathological, and molecular variables in a LASSO-penalized Cox model with 10-fold cross-validation. The optimal lambda value was selected as the one within 1 standard deviation of the minimum cross-validation error.

### 4.12. Statistical Analysis

The Spearman correlation was used to evaluate the relationship between variables. The Kruskal–Wallis and Fisher’s exact (with Benjamini–Hochberg correction were applicable) tests were used for comparisons between categorical variables. All data met the assumptions of the statistical tests used and were tested for normality. Statistical significance was set at *p* < 0.05 or FDR < 0.05. The analyses were performed using R language (v4.3.1) [90].

### 4.13. Limitations of the Study

Although the retrospective design and inclusion of surgically treated patients may limit generalizability, this approach reflects current standard-of-care and represents the most feasible methodology for studying rare malignancies such as ChS. The used tissue microarray (TMA) approach, while standardized, provides limited sampling that may not capture complete intratumoral heterogeneity and may provide bias in immunophenotyping, whereas the single staining IHC did not allow colocalization of immune cell markers. Therefore, in the future, it would be valuable to perform an analysis using multiplex techniques. The absence of matched normal controls prevents definitive somatic variant confirmation. In addition, due to the FFPE origin of the tissue, in a few samples we noticed an increased deamination score, which could influence the TMB value and variant calling.

## Data Availability

Raw sequencing data have been deposited in the European Nucleotide Archive (ENA) Genome Sequence Archive (https://www.ebi.ac.uk/ena/browser/home, accessed on 16 February 2026) with accession number PRJEB79550. Script for bioinformatic analysis of data from targeted next-generation sequencing of DNA samples has been deposited in Repository for Open Data—RepOD (https://doi.org/10.18150/YCIRA1, accessed on 16 February 2026). Any additional information required to reanalyze the data reported in this paper is available upon request.

## Supplementary Materials

The following supporting information can be downloaded at: https://www.mdpi.com/article/10.3390/ijms27042018/s1.

## Abbreviations

The following abbreviations are used in this manuscript:

Amp	amplifications of chromosomal region
APC	antigen-presenting cells
ChS	chondrosarcoma
DC	dendritic cell
DD/DDCS	dedifferentiated chondrosarcoma
Del	large-scale deletion
DFS	disease-free survival
Foxp3	Forkhead box P3
G	grade
Gal-9	galectin 9
iDC	immature dendritic cell
ICIs	immune checkpoint inhibitors
ICPs	immune checkpoints
IDH1	isocitrate dehydrogenase
IMP	immunophenotype
LAG-3	lymphocyte activation gene-3
LAMP3	lysosome-associated membrane glycoprotein
MAPK	mitogen-activated protein kinase
MSI	microsatellite instability
mTOR	mammalian target of rapamycin
Multi-hit	genes with more than one variant type
mut	mutated
NF-κB	nuclear factor kappa-light-chain-enhancer of activated B cells
NOTCH	neurogenic locus notch homolog protein
ns	non-significant
OS	overall survival
PCA	principal component analysis
PD-1	programmed death receptor 1
PD-L1	programmed death ligand 1
PI3K	phosphoinositide 3-kinases
RTK	receptor tyrosine kinases
SWI/SNF	SWItch/Sucrose Non-Fermentable
TAM	tumor-associated macrophage
Tc	cytotoxic T cells
TGFβ	transforming growth factor beta
Th	helper T cells
TIL	tumor infiltrating lymphocyte
TIM-3	T cell immunoglobulin and mucin domain-3
TLS	tertiary lymphoid structures
TMB	tumor mutation burden
Tregs	regulatory T cells
wt	wild type
