# Supplementary material for: Integrated Immune and Molecular Profiling Identifies Prognostic Subgroups and Therapeutic Targets in Chondrosarcoma
Source: Int J Mol Sci. 2026 Feb 20;27(4):2018. doi: 10.3390/ijms27042018 (PMC12940788; doi:10.3390/ijms27042018)
Supplement: Supplementary file 1 [file ijms-27-02018-s001.zip › Supplement.pdf]

Supplementary Tables

Table S1. Details of antibodies used for immunohistochemistry.

	Antibody	Company	Dilution	pH	Type of reaction	Detection system
1	CD45, clone 2B11+PD7/26	Agilent Technologies, Inc., Santa Clara, CA, USA	RTU	9	m	EnVision Flex +, Mouse High ph (link); K8002; Agilent Technologies, Inc.
2	CD3, clone F7.2.38	Agilent Technologies, Inc., Santa Clara, CA, USA	RTU	9	m	EnVision Flex +, Mouse High ph (link); K8002; Agilent Technologies, Inc
3	CD4, clone 4B12	Agilent Technologies, Inc., Santa Clara, CA, USA	RTU	9	m	EnVision Flex +, Mouse High ph (link); K8002; Agilent Technologies, Inc
4	CD8, clone C8/144B	Agilent Technologies, Inc., Santa Clara, CA, USA	RTU	9	m	EnVision Flex +, Mouse High ph (link); K8002; Agilent Technologies, Inc
5	CD68, clone PG-M1	Agilent Technologies, Inc., Santa Clara, CA, USA	RTU	9	c	EnVision Flex +, Mouse High ph (link); K8002; Agilent Technologies, Inc
6	CD68, clone KP1	Agilent Technologies, Inc., Santa Clara, CA, USA	RTU	9	c	EnVision Flex +, Mouse High ph (link); K8002; Agilent Technologies, Inc
7	CD163, clone MRQ-26	Roche Diagnostics International Ltd., Rotkreuz, Switzerland	RTU	V	c	UltraView Universal DAB Detection Kit; 760-500; Roche Diagnostics International Ltd.
8	PD-1, clone NAT105	Roche Diagnostics International Ltd., Rotkreuz, Switzerland	RTU	V	c	UltraView Universal DAB Detection Kit; 760-500; Roche Diagnostics International Ltd.
9	PD-L1, clone 22C3	Agilent Technologies, Inc., Santa Clara, CA, USA	RTU	6	m	System detekcyjny EnVision Flex +, Mouse High ph (link); K8002; Agilent Technologies, Inc.
10	Foxp3, clone EP340	Cell Marque™, Rocklin, CA, USA	1:200	9	n	UltraView Universal DAB Detection Kit; 760-500; Roche Diagnostics International Ltd.
11	CD14, clone EPR3653	Roche Diagnostics International Ltd., Rotkreuz, Switzerland	RTU	V	c	UltraView Universal DAB Detection Kit; 760-500; Roche Diagnostics International Ltd.
12	CD1a, clone 010	Agilent Technologies, Inc., Santa Clara, CA, USA	RTU	9	c	EnVision Flex +, Mouse High ph (link); K8002; Agilent Technologies, Inc
13	CD141, clone 141C01	LifeSpan BioSciences, Inc., Lynnwood, Washington, D.C., USA	1:50	6	m	EnVision Flex +, Mouse High ph (link); K8002; Agilent Technologies, Inc

	Antibody	Company	Dilution	pH	Type of reaction	Detection system
14	CD80, clone OTI2E5	LifeSpan BioSciences, Inc., Lynnwood, Washington, D.C., USA	1:50	6	c/m	EnVision Flex +, Mouse High ph (link); K8002; Agilent Technologies, Inc
15	LAG-3, clone D2G4O	Cell Signaling Technology, Inc., Danvers, MA, USA	1:50	9	m	EnVision Flex +, Mouse High ph (link); K8002; Agilent Technologies, Inc
16	TIM-3, clone D5D5R	Cell Signaling Technology, Inc., Danvers, MA, USA	1:100	9	n	EnVision Flex +, Mouse High ph (link); K8002; Agilent Technologies, Inc
17	Galectin 9, clone D9R4A	Cell Signaling Technology, Inc., Danvers, MA, USA	1:300	9	c/m	EnVision Flex +, Mouse High ph (link); K8002; Agilent Technologies, Inc
18	HLA-DR, clone TAL1B5	Santa Cruz Biotechnology, Inc., Dallas, TX, USA	1:300	6	c/m	EnVision Flex +, Mouse High ph (link); K8002; Agilent Technologies, Inc
19	CD20, clone L26	Agilent Technologies, Inc., Santa Clara, CA, USA	RTU	9	m	EnVision Flex +, Mouse High ph (link); K8002; Agilent Technologies, Inc
20	LAMP3	Thermo Fisher Scientific Inc., Waltham, MA, USA	1:100	9	m	EnVision Flex +, Mouse High ph (link); K8002; Agilent Technologies, Inc

Abbreviations: c—cytoplasmic, DAB—3,3'-Diaminobenzidine, m—membranous, n—nuclear, RTU—ready-to-use, V—Ventana platform.

Table S2. Characterization of immune markers within the central and peripheral region of the tumor.

Marker	Cell type with marker expression	Mean density in center	Mean density in periphery	Median density in center	Median density in periphery	p-value	FDR
CD14	monocyte	179.57	259.03	56.51	108.07	0.027	0.0733
CD141	DC	5.01	31.20	0.00	0.00	<0.0001	**0.0002**
CD163	M2-like TAM	172.71	128.43	85.40	70.68	0.649	0.7987
CD1a	iDC	0.95	3.50	0.00	0.00	0.0260	0.0733
CD20	B cell	24.13	17.74	0.00	0.00	0.9586	0.9586
CD3	T cell	23.21	24.70	0.00	5.33	0.0159	0.0603
CD4	Th	7.21	24.7	0.00	1.61	<0.0001	**0.0008**
CD45	leukocyte	94.60	148.70	9.54	34.03	0.0066	**0.0313**
CD68 [KP1]	M2-like TAM	63.34	67.21	21.12	17.58	0.8564	0.9413
CD68 [PGM1]	M1-like TAM	166.45	127.78	58.10	70.68	0.8918	0.9413
CD8	Tc	20.25	19.06	0.00	3.09	0.3713	0.5609
CD80	APC	0.41	1.02	0.00	0.00	0.2300	0.4012
Foxp3	Treg	3.30	5.77	0.00	0.00	0.0817	0.1941
Gal-9	TIL, APC, tumor cell	297.28	313.01	16.69	50.57	0.5904	0.7987
HLA-DR	APC, tumor cell	447.97	517.22	112.07	133.53	0.1448	0.3057
LAG-3	TIL	23.65	36.90	0.00	0.00	0.3838	0.5609
LAMP3	DC	0.20	1.30	0.00	0.00	0.6726	0.7987
PD-1	TIL	0.40	1.27	0.00	0.00	0.0003	**0.0016**
TIM-3	TIL	86.50	92.51	0.00	0.00	0.2322	0.4012

Bold font indicates statistically significant results (FDR<0.05). For comparisons of densities of individual markers in the central and peripheral regions of the tumor, the Wilcoxon test for paired observations was used. Abbreviations: APC—antigen-presenting cells, DC—dendritic cell, FDR—false discovery rate, calculated using Benjamini-Hochberg procedure, Foxp3—Forkhead box P3, Gal-9—galectin 9, iDC—immature dendritic cell, LAG-3—lymphocyte activation gene-3, LAMP3—lysosome-associated membrane glycoprotein, PD-1—programmed death receptor 1, PD-L1—programmed death ligand 1, TAM—tumor-associated macrophage, Tc—cytotoxic T cells, Th—helper T cells, TIL—tumor infiltrating lymphocyte, TIM-3—T cell immunoglobulin and mucin domain-3, Tregs—regulatory T cells.

Table S3. Fisher exact test results of testing the differences of gene mutations across tumor subtypes and immune clusters.

Across tumor subtypes			***Across immune clusters***		
**Gene**	**p-value**	**FDR**	**Gene**	**p-value**	**FDR**
TP53	0.0000	**0.0011**	*IDH2*	0.0030	0.1143
IDH2	0.0001	**0.0046**	*IDH1*	0.0162	0.6000
IDH1	0.0004	**0.0138**	*PTCH1*	0.0217	0.7812
PTCH1	0.0098	0.3439	*RET*	0.0988	1.0000
AKT2	0.0255	0.8673	*SYNE1*	0.8976	1.0000
RET	0.7706	1.0000	*USP9X*	0.5034	1.0000
SYNE1	0.3798	1.0000	*BCL11B*	0.8105	1.0000
USP9X	0.3299	1.0000	*KDM5C*	0.6675	1.0000
BCL11B	1.0000	1.0000	*MN1*	0.2193	1.0000
KDM5C	0.6543	1.0000	*MYH9*	0.1522	1.0000
MN1	0.1124	1.0000	*ADGRA2*	0.4332	1.0000
MYH9	0.7080	1.0000	*AKT2*	0.0949	1.0000
ADGRA2	1.0000	1.0000	*ARID1A*	0.3789	1.0000
ARID1A	0.3299	1.0000	*ATRX*	0.4842	1.0000
ATRX	0.9455	1.0000	*BCR*	1.0000	1.0000
BCR	1.0000	1.0000	*CREBBP*	1.0000	1.0000
CREBBP	0.7208	1.0000	*CSMD3*	0.1843	1.0000
CSMD3	0.1618	1.0000	*DNMT3A*	0.5018	1.0000
DNMT3A	0.9455	1.0000	*EPHA7*	0.0484	1.0000
EPHA7	0.1075	1.0000	*EXT2*	1.0000	1.0000
EXT2	0.1944	1.0000	*FGFR3*	0.6219	1.0000
FGFR3	0.7080	1.0000	*KMT2A*	0.6219	1.0000
KMT2A	0.2930	1.0000	*KMT2C*	0.6219	1.0000
KMT2C	0.3110	1.0000	*LRP1B*	0.5848	1.0000
LRP1B	0.5006	1.0000	*MYH11*	0.2172	1.0000
MYH11	0.6540	1.0000	*NOTCH1*	1.0000	1.0000
NOTCH1	0.5660	1.0000	*NOTCH2*	0.1515	1.0000
NOTCH2	0.4179	1.0000	*NUP214*	0.0865	1.0000
NUP214	0.7700	1.0000	*PTPRT*	0.7630	1.0000
PTPRT	0.2516	1.0000	*RB1*	0.5148	1.0000
RB1	0.3488	1.0000	*RNF213*	0.5137	1.0000
RNF213	0.8779	1.0000	*SETD2*	0.1522	1.0000
SETD2	0.6742	1.0000	*STK11*	0.6219	1.0000
STK11	0.6071	1.0000	*TAF1L*	0.3696	1.0000
TAF1L	0.4613	1.0000	*TP53*	0.0364	1.0000
TRRAP	0.2930	1.0000	*TRRAP*	1.0000	1.0000
UBR5	0.1022	1.0000	*UBR5*	0.2487	1.0000
TAF1	0.8779	1.0000	*TAF1*	0.3956	1.0000

Abbreviations: FDR—false discovery rate. Bold font indicates statistically significant results (FDR <0.05). The table presents results for gene mutation present in at least 5 patients. Fisher exact test was used for the comparisons between groups.

Table S4. Univariate and multivariate analyses of the impact of clinical factors on patients' survival.

			OS		DFS	
Feature	*Level*	*n*	*HR univariable (95% CI; p-value)*	*HR multivariable (95% CI; p-value*	*HR univariable (95% CI; p-value)*	*HR multivariable (95% CI; p-value*
Gender[a]	Female	42	-	-	-	-
	Male	57	1.24 (0.66-2.35; p=0.504)	-	1.16 (0.68-1.98; p=0.594)	-
Age at diagnosis-per 1 year change	-	-	1.04 (1.02-1.05; **p<0.001**)	1.02 (1.00-1.05; **p=0.037**)	1.03 (1.01-1.04; **p<0.001**)	1.02 (1.00-1.04; p=0.056)
Tumor size - per 1 mm change	-	-	1.01 (1.01-1.01; **p<0.001**)	1.01 (1.00-1.01; **p=0.001**)	1.01 (1.00-1.01; **p<0.001**)	1.01 (1.00-1.01; **p=0.007**)
Pathological Fracture	No	90	-	-	-	-
	Yes	9	2.59 (1.19-5.62; **p=0.016**)	1.51 (0.66-3.46; p=0.328)	2.28 (1.08-4.83; **p=0.032**)	2.14 (0.92-4.95; p=0.077)
Tumor localization	Axial	35	-	-	-	-
	Lower limb	28	4.56 (1.87-11.13; **p=0.001**)	2.48 (0.89-6.87; p=0.081)	2.72 (1.38-5.38; **p=0.004**)	2.27 (1.06-4.89; **p=0.036**)
	Upper limb	36	3.40 (1.41-8.16; **p=0.006**)	1.99 (0.76-5.26; p=0.164)	2.00 (1.04-3.84; **p=0.038**)	1.19 (0.54-2.62; p=0.666)
Histological subtype	G1	28	0.04 (0.01-0.13; **p<0.001**)	0.16 (0.04-0.66; **p=0.011**)	0.03 (0.01-0.09; **p<0.001**)	0.08 (0.02-0.28; **p<0.001**)
	G2	37	0.10 (0.04-0.25; **p<0.001**)	0.27 (0.09-0.80; **p=0.018**)	0.09 (0.04-0.21; **p<0.001**)	0.19 (0.07-0.51; **p=0.001**)
	G3	24	0.28 (0.12-0.66; **p=0.004**)	0.59 (0.21-1.65; p=0.315)	0.22 (0.09-0.52; **p=0.001**)	0.43 (0.16-1.17; p=0.099)
	DD	10	-	-	-	-
Resection margin	R0	79	-	-	-	-
	R1	11	1.08 (0.45-2.59; p=0.867)	-	0.99 (0.46-2.11; p=0.979)	-
	R2	9	0.76 (0.23-2.50; p=0.657)	-	0.77 (0.27-2.13; p=0.610)	-

[a] assigned based on medical reports. Bold font indicates statistically significant results (p<0.05). Abbreviations: CI—confidence interval, DD—dedifferentiated chondrosarcoma, DFS—disease-free survival, HR—hazard ratio, n—number of patients, OS—overall survival.

Table S5. Univariate analysis of the impact of mutated genes on patients' overall survival.

Gene	HR	CI (LL)	CI (UL)	p-value	FDR
IDH1	1.2528	0.6239	1.8817	0.0001	**0.0036**
TP53	1.3545	0.6416	2.0676	0.0002	**0.0037**
EPHA7	1.8074	0.7244	2.8904	0.0011	**0.0136**
PTCH1	1.7093	0.6271	2.7913	0.0020	**0.0186**
UBR5	1.3201	0.2660	2.3742	0.0141	0.1072
IDH2	0.9229	0.1367	1.7092	0.0214	0.1356
RET	1.0525	0.0161	2.0890	0.0466	0.2243
MN1	0.9645	0.0119	1.9172	0.0472	0.2243
ARID1A	1.0134	-0.0233	2.0501	0.0554	0.2339
RNF213	-1.8397	-3.8247	0.1453	0.0693	0.2633
EXT2	1.2738	-0.1862	2.7338	0.0873	0.2815
MYH9	1.0421	-0.1585	2.2427	0.0889	0.2815
AKT2	0.8529	-0.1817	1.8874	0.1061	0.3103
STK11	-1.1362	-3.1232	0.8508	0.2624	0.6108
CREBBP	0.6785	-0.5112	1.8682	0.2637	0.6108
PTPRT	-1.1260	-3.1113	0.8593	0.2663	0.6108
KDM5C	0.4977	-0.4408	1.4362	0.2986	0.6108
ADGRA2	-1.0503	-3.0371	0.9365	0.3001	0.6108
KMT2A	-1.0340	-3.0212	0.9532	0.3078	0.6108
RB1	0.6010	-0.5871	1.7892	0.3215	0.6108
LRP1B	-0.5785	-1.7660	0.6089	0.3396	0.6146
NOTCH1	-0.9278	-2.9132	1.0576	0.3597	0.6193
TAF1L	0.4648	-0.5702	1.4999	0.3788	0.6193
SYNE1	0.4098	-0.5269	1.3465	0.3912	0.6193
USP9X	-0.7795	-2.7658	1.2069	0.4418	0.6716
KMT2C	0.4072	-0.7698	1.5843	0.4977	0.7274
ATRX	-0.4381	-1.8599	0.9836	0.5458	0.7682
BCR	0.3098	-0.8701	1.4898	0.6068	0.7986
TAF1	-0.2691	-1.3016	0.7634	0.6095	0.7986
BCL11B	-0.2066	-1.2416	0.8285	0.6957	0.8644
MYH11	0.2754	-1.1513	1.7021	0.7052	0.8644
NUP214	-0.2209	-1.6444	1.2026	0.7610	0.8656
DNMT3A	0.1587	-0.8737	1.1911	0.7633	0.8656
TRRAP	-0.2081	-1.6314	1.2153	0.7745	0.8656
SETD2	0.1309	-1.0468	1.3087	0.8275	0.8985
NOTCH2	0.0605	-1.1161	1.2370	0.9198	0.9622
FGFR3	-0.0576	-1.4833	1.3681	0.9369	0.9622
CSMD3	-18.1405	-9076.1309	9039.8499	0.9969	0.9969

Abbreviations: CI—confidence interval, FDR—false discovery rate, LL—lower limit, n—number of patients with mutated gene, UL—upper limit. Bold font indicates statistically significant results (FDR <0.05). The table presents results for n > 5.

Table S6. Patients' characteristics.

Feature	Level	Value
N	-	99
Gender[a] – n; %	Female	42; 42.4
	Male	57; 57.6
Age at diagnosis – mean (SD) [years]	-	53.00 (+/-17.13)
Tumor size – mean (SD) [mm]	-	122.64 (+/- 65.83)
Histological subtype - n; %	G1	28; 28.3
	G2	37; 37.4
	G3	24; 24.2
	Dedifferentiated	10; 10.1
Tumor localization - n; %	Axial	35; 35.4
	Lower limb	28; 28.3
	Upper limb	36; 36.4
Resection morbidity - n; %	Local resection	64; 64.6
	Resection with prosthesis implantation	17; 17.2
	Amputation	18; 18.2
Resection margin - n; %	R0	79; 79.8
	R1	11; 11.1
	R2	9; 9.1
Resection type - n; %	Excisional biopsy	3; 3.0
	Tumor resection	90; 90.9
	Whoops operation	6; 6.1
Local relapse - n; %	Yes	28; 28.9
	No	69; 71.1
Distant metastasis - n; %	Yes	28; 28.9
	No	69; 71.1
Pathological Fracture n; %	Yes	9, 9.1
	No	90; 90.9

[a] assigned based on medical report. Abbreviations: n—number of patients, SD—standard deviation.

Table S7. Summary statistics of adjusted Rand index (ARI) values obtained from bootstrap resampling assessing the stability of the identified immunopthenotypes (IMPs).

**Statistic**	**ARI**
Minimum	0.02
1st quartile	0.30
Median	0.41
Mean	0.44
3rd quartile	0.56
Maximum	1.00

Bootstrap resampling demonstrated moderate stability of the three IMPs, with a median ARI of 0.41 (mean 0.44, interquartile range 0.30–0.56). This indicates a reproducible global clustering structure with some variability in individual case assignment across resampled datasets.

Supplementary Figures

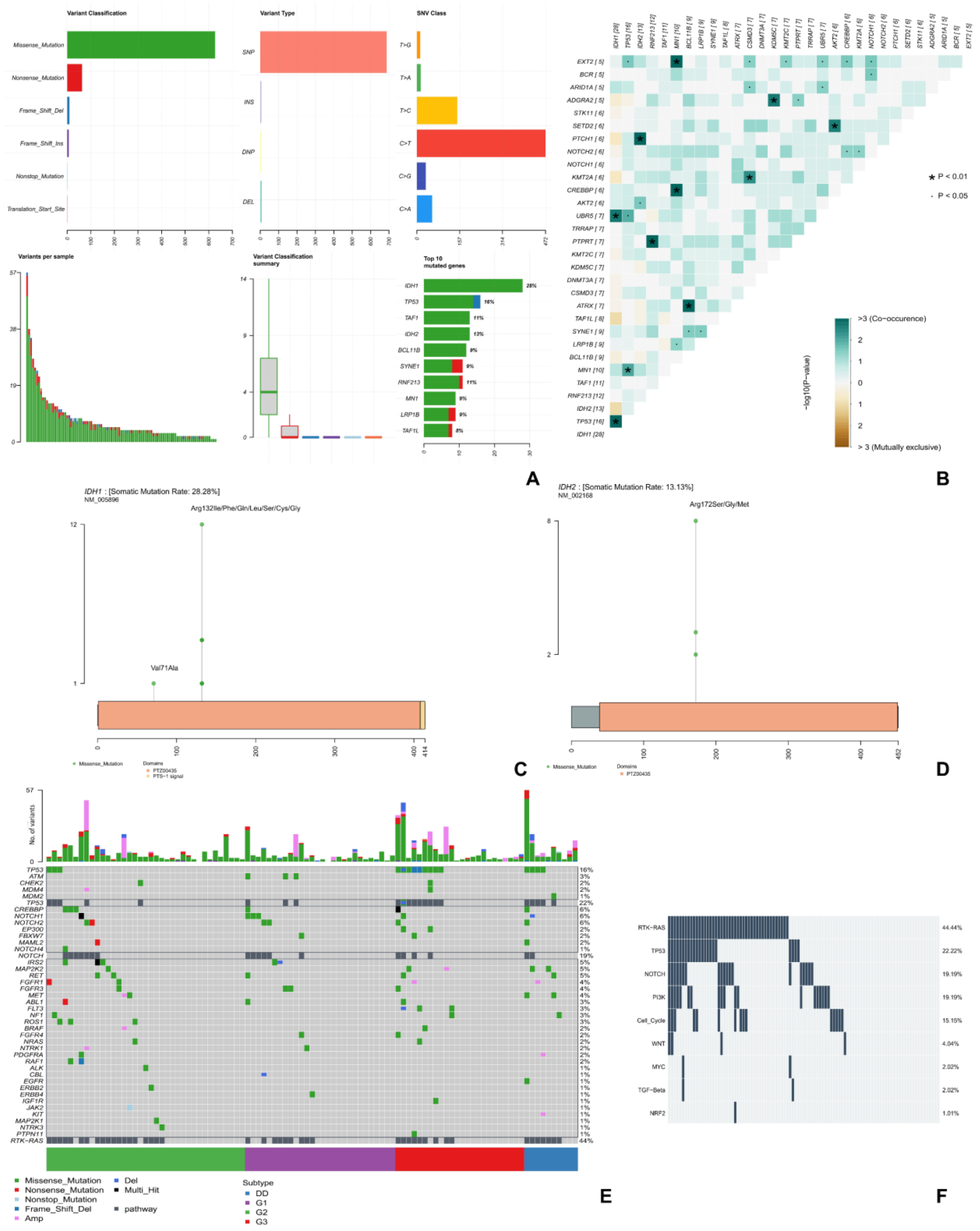

Figure S1. Detailed data of molecular variants and alternative pathway analysis.

A - Summary of single-nucleotide variants and INDELs across 99 patients with chondrosarcoma. B - Correlation matrix of the 30 most frequently mutated genes. Fisher's exact test was used for comparisons between groups. C, D - Number and localization of amino acid changes found in *IDH1* (left) and *IDH2* (right) genes. E - Characterization of the 3 top deregulated pathways in the study population, with their frequency among patients and including histological subtypes. The classification

was based on Sanchez-Vega et al.[89] F - Characterization of all deregulated pathways and their frequency across the study population based on Sanchez-Vega et al.[89]. Abbreviations: Amp—amplifications of chromosomal region, Complex event—identifies both single-nucleotide variants and genetic aberration, DD—dedifferentiated chondrosarcoma. Del—large-scale deletion, IDH—Isocitrate Dehydrogenase, Multi-hit—genes with more than one variant type, Multi-hit—genes with more than one variant type, mut—mutated, NOTCH—neurogenic locus notch homolog protein, PI3K—phosphoinositide 3-kinases, RTK—receptor tyrosine kinases, TGFβ—transforming growth factor beta.

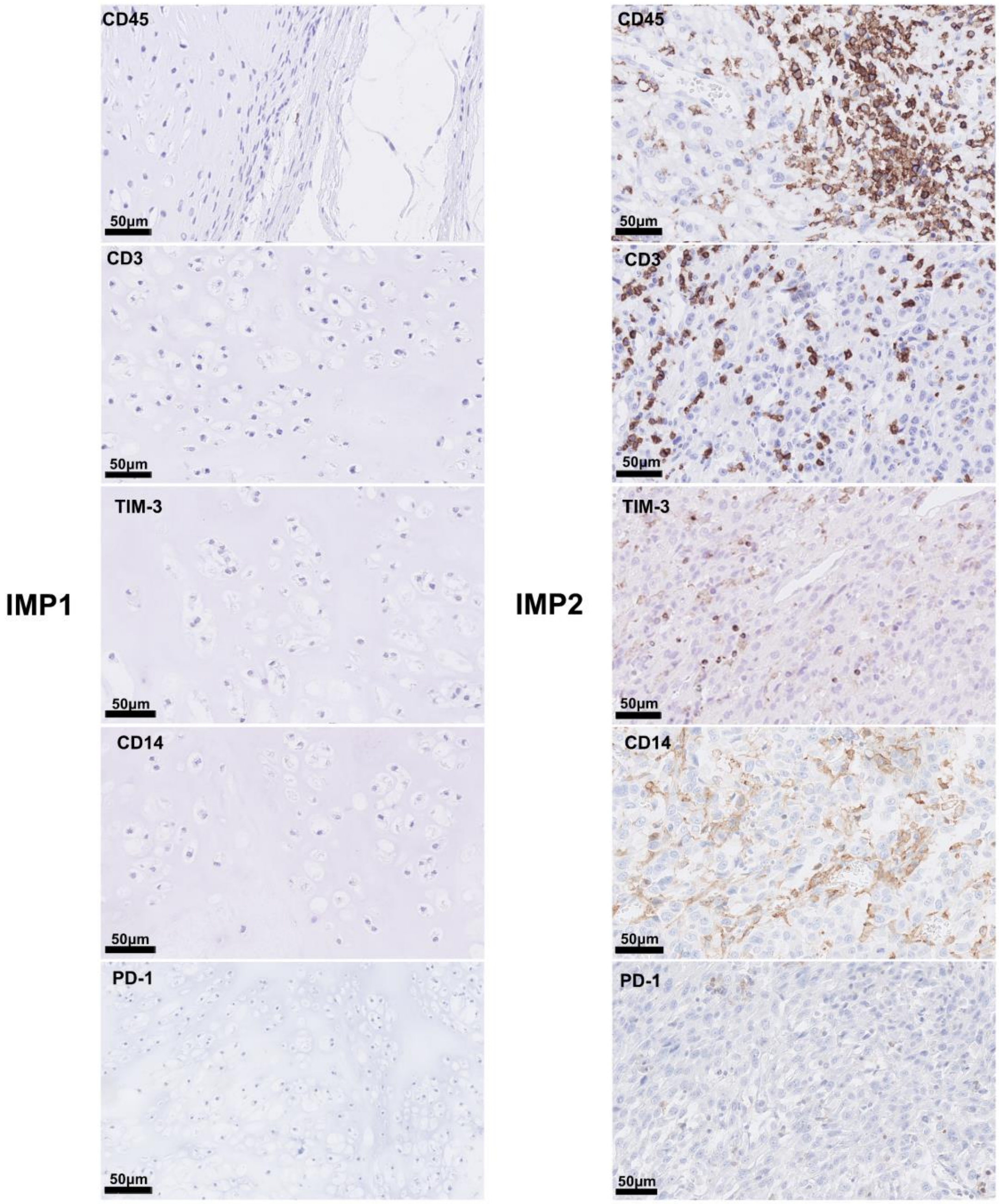

Figure S2. The examples of immunohistochemical staining of selected markers for patients with IMP1 (“cold”) and IMP2 (“hot”).

IMP1 was characterized by low expression of immune markers, whereas IMP2 was characterized by high expression of these markers. The images show the peripheral region of the tumor for CD45 expression and the central regions of the tumor for the rest of the markers. The images present additional selected markers with the highest impact across different IMPs. Abbreviations: IMP—immunophenotype, PD-1—programmed death receptor 1, TIM-3—T cell immunoglobulin and mucin domain-3.

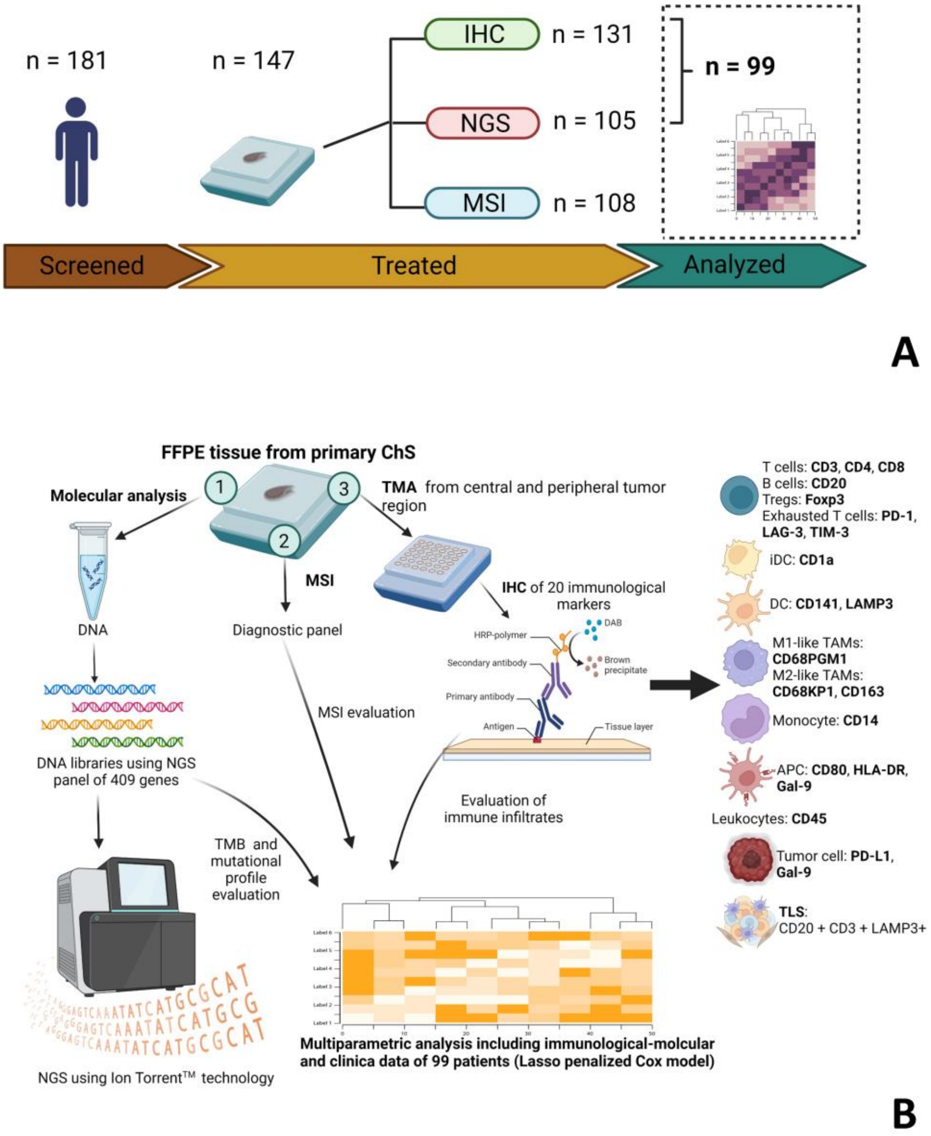

Figure S3. Study design.

A - Flowchart of patient enrollment. B - Research scheme. Created with BioRender.com. Abbreviations: IHC—immunohistochemistry, MSI—microsatellite instability, NGS—next-generation sequencing, TMA—tissue microarrays, TMB—tumor mutation burden, TME—tumor microenvironment.

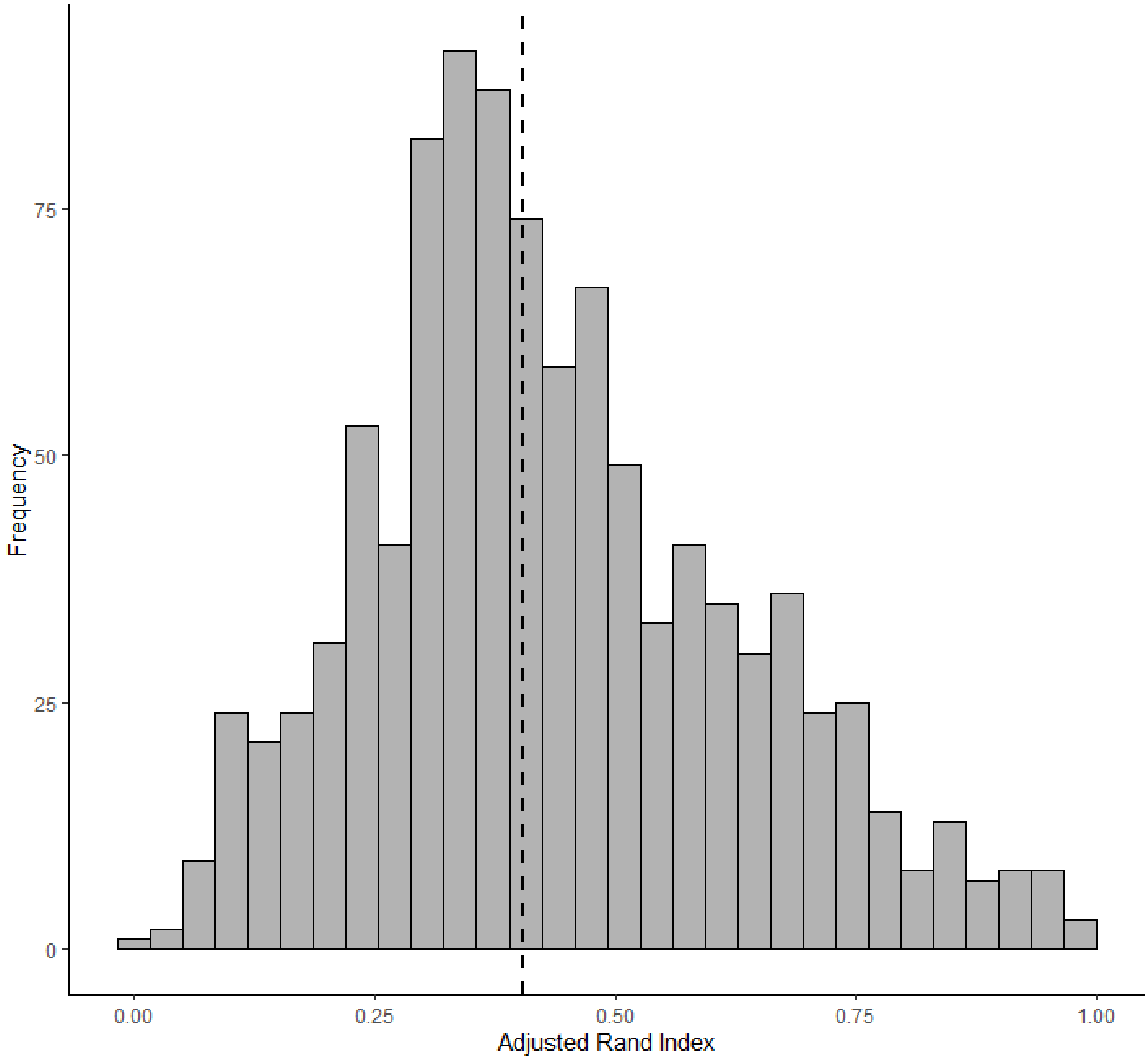

Figures S4. Histogram of adjusted Rand index (ARI) values from 1,000 bootstrap resampling iterations evaluating the stability of the three identified clusters - immunophenotypes (IMPs). ARI median value (0.41) has been indicated by the dashed line.
